# Highly-sensitive capture of circulating tumor cells using micro-ellipse filters

**DOI:** 10.1038/s41598-017-00232-6

**Published:** 2017-04-04

**Authors:** Hongmei Chen, Baoshan Cao, Bo Sun, Yapeng Cao, Ke Yang, Yu-Sheng Lin

**Affiliations:** 1 Institute of Semiconductors, Chinese Academy of Sciences, Beijing, 100083 China; 2 Division of Nanobionic Research, Suzhou Institute of Nano-Tech and Nano-Bionics, Chinese Academy of Sciences, Suzhou, Jiangsu 215123 China; 3 Department of chemotherapy and radiation sickness, Peking University Third Hospital, Beijing, 100191 China; 4 Physics Department, University of Massachusetts Lowell, Lowell, Massachusetts 01854 USA

## Abstract

Circulating tumor cells (CTCs) detection, enumeration and characterization with microfluidic chips has critical significance in cancer prognosis offering a non-invasive “liquid biopsy”. Based on physical differences of size and deformability, we explore micro-ellipse filters consisting of microfuidic slits in series gradually narrowed. Slender tunnels sensitively capture tumor cells with slim chance to escape. Tumor cells could reside at capture sites organized by arrays of micro-ellipse microposts enduring less stress. Circular elliptical microstructures produce smooth flow minimally reducing any damage. “Air Suction” could extremely shorten capture. Capture efficiency comes out to be a robust yield of 90% and percentage obeys Gaussian distribution at various stages. With rare number accurately enumerated, micro-Ellipse filters have been tested high efficiently capturing tumor cells in both whole and lysed blood. To clinically validate the device, the microfluidic chip was utilized to identify and capture CTCs from metastatic breast, colon and non-small-cell lung (NSCLC) cancer patients. CTCs were detected positive in all samples with 4 patients having more than 20 CTCs. Those sensitive results are consistent with theoretical expectation. Efficient micro-ellipse filters enable clinical enumeration of metastasis, on-chip anti-cancer drug responses and biological molecular analysis.

## Introduction

Circulating tumor cells (CTCs)^1^ enter blood stream, causing metastatic spread and growth of tumor cells at distant sites^2^. Metastasis is decisive for life of cancer patients^3,4^. Number of CTCs is related to overall progression-free survival^5^ since more CTCs indicate shorter survival^6^. Enumeration of CTCs is the key to evaluate disease severity^6,7^ and explore biologic mechanism of metastasis. However, only 1–100 CTCs among billions of normal cells^8^, this poses high technique challenge. Currently, Veridex Cellsearch system (Raritan, NJ, USA) is only FDA-approved for successfully clinical enumeration of breast, prostate and colorectal cancers through immunomagnetic technology^9,10^. Microfluidic chips appear as an attractive alternative to conventional biochemical methods offering a “liquid biopsy”^11^ utilizing immune-affinity, physical separation and immuomagnetic approaches. CTC-chip^12^, Herringbone-chip (HB-Chip)^13^, patterning regions of alternating adhesive proteins^14^, a grapheme oxide chip^15^, a 3D graphene oxide microchip^16^, OncoBean chip^17^ and a GO-polymer device^18^ realize affinity-based isolation. The abovementioned approaches resort to a specific antigen, EpCAM or aptamers^19,20^. Due to epithelial-to-mesenchymal transition (EMT)^21^, metastasis changes epithelial characteristic of tumor cells into the mesenchymal in the invasion, causing EpCAM loss^22^. Hence, physical separation of size- and deformability-based filtration is widely adopted in the CTCs isolation, owing to CTCs being larger and less deformable than normal blood cells^23^. 8 µm has been proved as a valuable and optimal size in retrieval of CTCs, a gold standard and cutoff pore size to filter^24^. Smaller size such as 5 µm or 4 µm also has been applied^25^. Slanted spiral microfluidics could ultra-fast, label-free isolate CTCs^26^ utilizing Vortex technology combining use of micro-scale vortices and inertial focusing^8^. A centrifugal-force-based size-selective CTC microfluidic device^27^, a CTC Cluster-Chip^28^, a cascaded spiral microfluidic device^29^, a microfluidic rachet^30^ and a multi-obstacle architecture (MOA) filter^31^ are recently described. Dedicated efforts have been made to overcome size-overlapped between CTCs (15 µm–25 µm) and White blood cells (WBCs) (9 µm–16 µm)^32^. Sensitivity and reproducibility still remain our aim to be improved. Some current microfluidic chips for CTCs isolation have been listed (See Supplementary Table 1).

**Figure 1:**
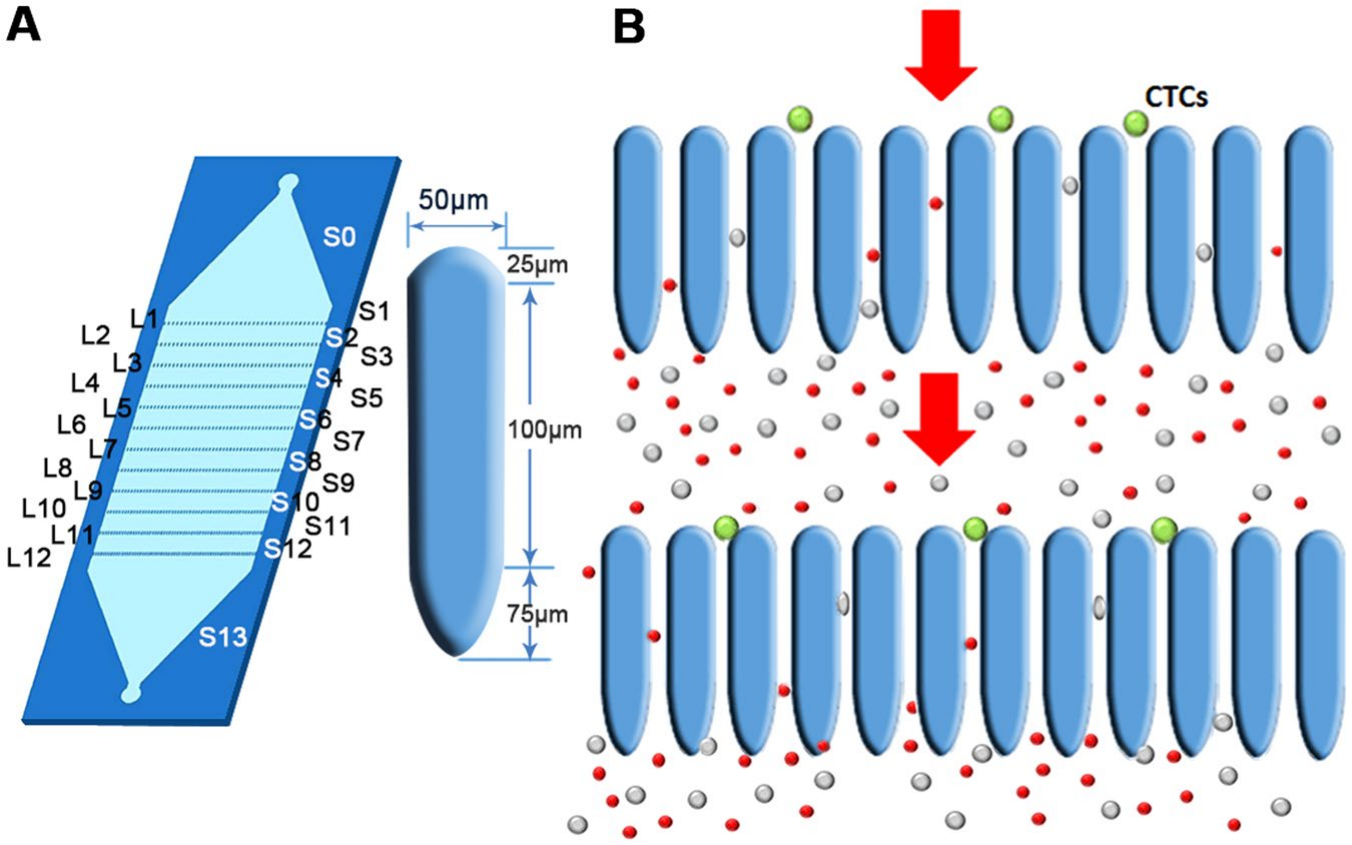
Configuration and operational diagram. (**A**) Diagram of Ellipse filters and configuration of single micro-elliptical pillar. Gap sizes of different arrays for micro-Ellipse filters ranging from L1 to L12 are18 µm, 16 µm, 14 µm, 12 µm, 9 µm, 9 µm, 7 µm, 7 µm, 5 µm, 5 µm, 5 µm, 5 µm, respectively. S0 to S13 are stages between two neighboring arrays. The distance between two successive arrays is 1500 µm. (**B**) Schematic of operating principle of CTC capture in front of Ellipse filters.

Here we introduce micro-ellipse filters (Ellipse filters) for highly-sensitive, robust and reproducible capture of CTCs. Successive micro-Ellipse filters in series ensure captured CTCs avoiding being flowed away by hydrodynamics forces. Filters have finer gap spacings organized by micro-elliptical microposts from upstream to downstream. A circularly elliptical micro-structure is designed to prevent any detrimental frequently met for square, rectangular^33^ or even weir-style physical barriers^34^ (Fig. 1). Smooth edges are frictionless and harmless and elongated passageways keep tumor cells from entry. This flowing structure with slim tunnels is pivotal to capture CTCs with high sensitivity and viability. The characteristic of this approach relies on physical differences of size and deformability between tumor cells and haematological cells. Quantitatively precise rare cells enumeration is feasible for artificial patient assays. Furthermore, performance of the device has been clinically validated through capturing CTCs from four metastatic breast cancer patients, one colon patient and twelve non-small-cell lung (NSCLC) cancer patients.

### Separation principle

**Figure 2:**
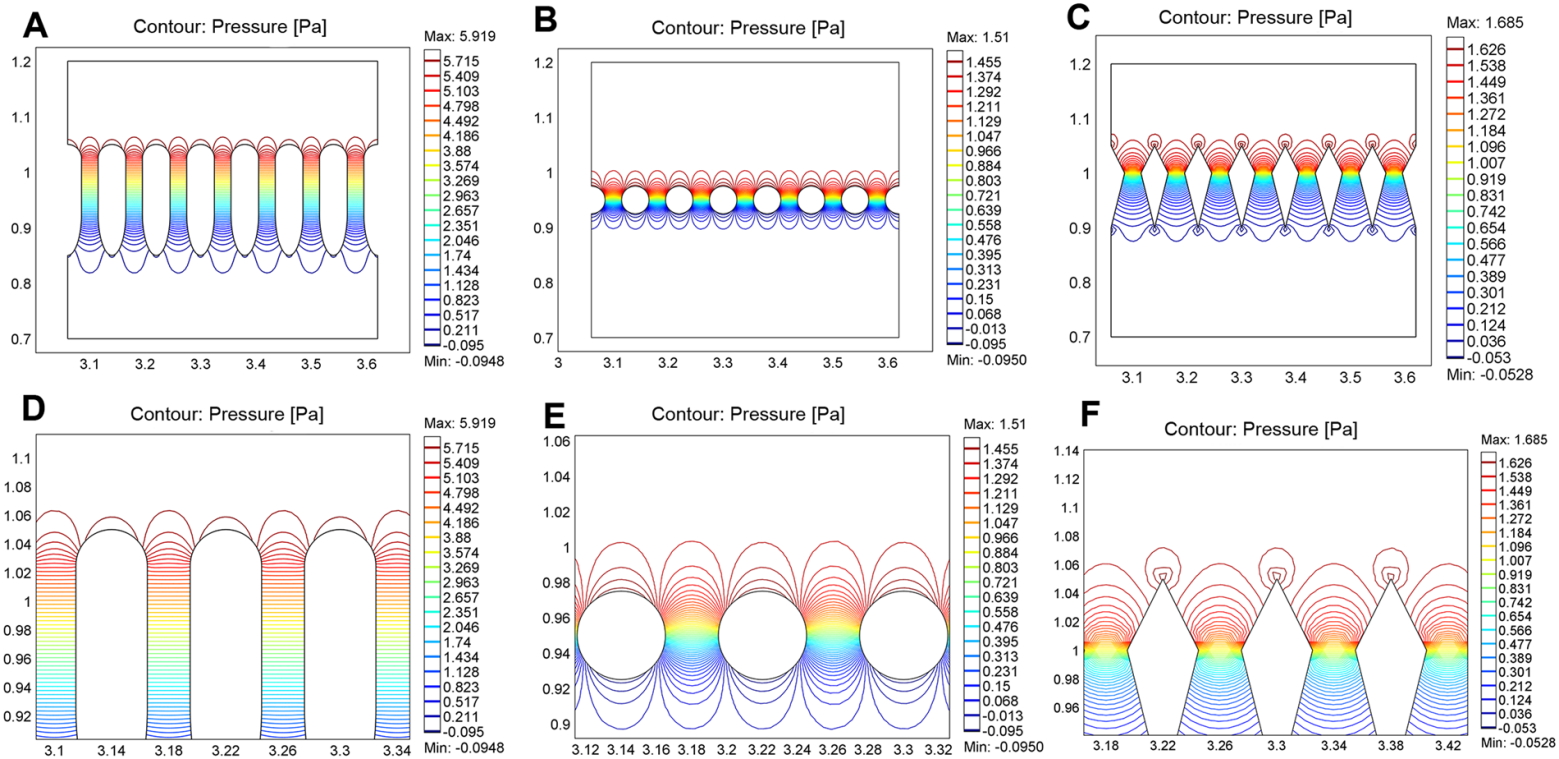
Comparison of pressure contours for three microfluidic chips composing of three distinct microposts. (**A**) Pressure contour for ellipse microposts array. (**B**) Pressure contour for circular microposts array. (**C**) Pressure contour for prismatic microposts array. (**D**) Pressure contour for magnified ellipse microposts array. (**E**) Pressure contour for magnified circular microposts array. (**F**) Pressure contour for magnified prismatic microposts array.

Micro-Ellipse filters could transport CTCs samples through a matrix of ellipse constriction, impeding large, stiff tumour cells and allowing small, malleable cells to transit through. 8 µm has been found to be a little wider for discriminating every CTCs (See Supplementary Fig. 1), thus we choose 5 µm as the narrowest gap spacing. The width between successive rows is 1500 µm and the depth of the device or the ceiling height of the microposts is 70 µm. There are totaling 12 arrays from L1 to L12 with gaps ranging from 18 µm to 5 µm, and each row has almost two hundred micro-ellipse pillars. S is the stage between two neighboring arrays. Big gap spacing was utilized to obstruct impurities, and small ones were used to segregate. The micro-ellipse is made up of three components, a half circle with radius 25 µm, a rectangular with length 100 µm and width 50 µm and a half ellipse with half long axis 75 µm. Dimension of the micro-ellipse and height is chosen comparably to several tumor cell sized rendering strengthen structure and smooth flow. Delicate slightness tunnels could prevent CTCs from entering, thus greatly promote sensitivity. Once CTCs are interrupted, CTCs recline on entrancing capture sites by microposts organization or transit between two adjacent arrays of micropillars, impossible to deform to passage through (Fig. 1). From simulation and mechanic analysis of filters composing of ellipse pillars, circular posts and prismatic ones (Fig. 2), we could see circular elliptical microstructures experiencing minimum pressure difference. Thus, ellipse filters would reduce friction and shear stress producing any damage greatly ensuring viability. It would have no impair on tumor cells with sharp angles. Slender tunnels ensure captured CTCs without being escaped except relaxed in front of micropost arrays. They function as barriers to CTCs yielding permission to small deformable blood constituents. Thus, those frictionless gradual micro-ellipse filters ensure high reproducible sensitivity. Distribution of distinct malignant cells is determined by their malignancy capability to pass across varied gradually narrowed microchannels. Clogging phenomenon could be extensively mitigated through red blood cells lysis (RBCL) followed by gently flushing to dislodge non-specific adhension of blood cells. Since broader regions exist between two adjacent arrays compares to length of the micropillar. Thus, CTCs mostly retain inside the media keeping viability enabling following biological molecular analysis. This fine designed constriction facilitates viability.

## Results and Discussion

### Optimal flow rate

**Figure 3:**
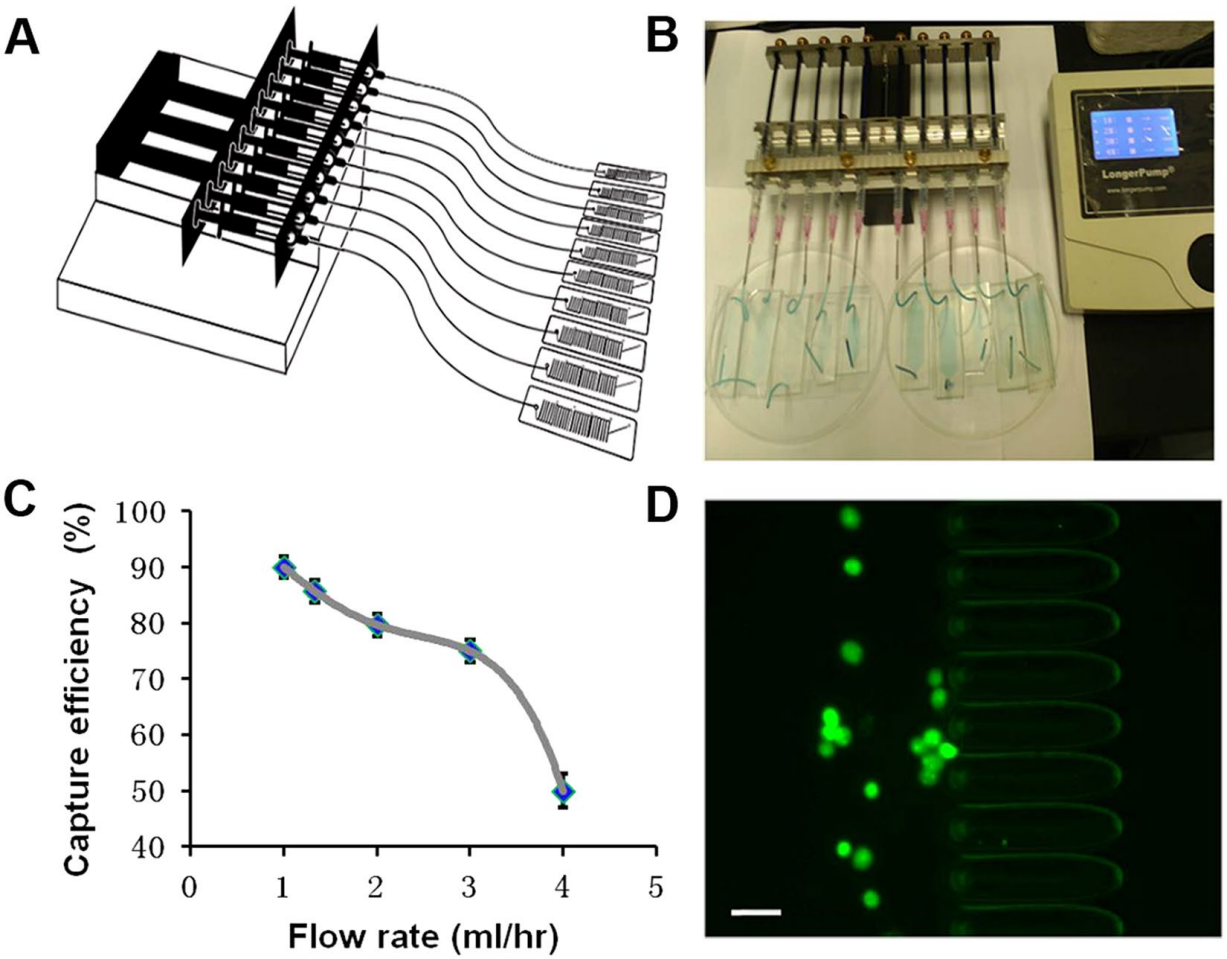
(**A**) Diagram of microfluidic chips connecting to a 10-channel syringe pump. (**B**) Photography of microfluidic system for ultra-sensitive capture of CTCs. (**C**) Capture efficiencies of Ellipse filters at varied flow rates. (**D**) Tumor cells are captured before the micropillars. Scale bar = 50 µm.

Capture efficiencies for Ellipse filters are evaluated at various flow rates (Fig. 3). We spiked 100 breast cancer cells (MCF-7) into phosphate buffered saline (PBS) and enumerated tumour cells captured on Ellipse filters and flowing out. Several identical comb-like filters in series with porosity narrowed down are effective in capturing. Low flow rates corresponding to high capture efficiency are preferrable, whereas high shear stress produced at high flow rates would do maleficence to cells. Extremely high hydrodynamic forces would release trapped cells. Also due to greatly enhanced flow rate, more malignant tumor cells tend to deform to squeeze out. In order to get rigorous, robust and reproducible capture efficiency and find each CTC in patient blood sample, we choose low flow rate as the optimal. In Fig. 3A, capture efficiency is better than 90% at 1 ml/h, and 80% still could be obtained at 2 ml/h. Therefore, subsequent assays were performed at the optimal flow rate of 1 ml/h. CTCs visually evidently appear in front of Ellipse filters shown in Fig. 3D.

### Isolation results and efficiency in PBS

**Figure 4:**
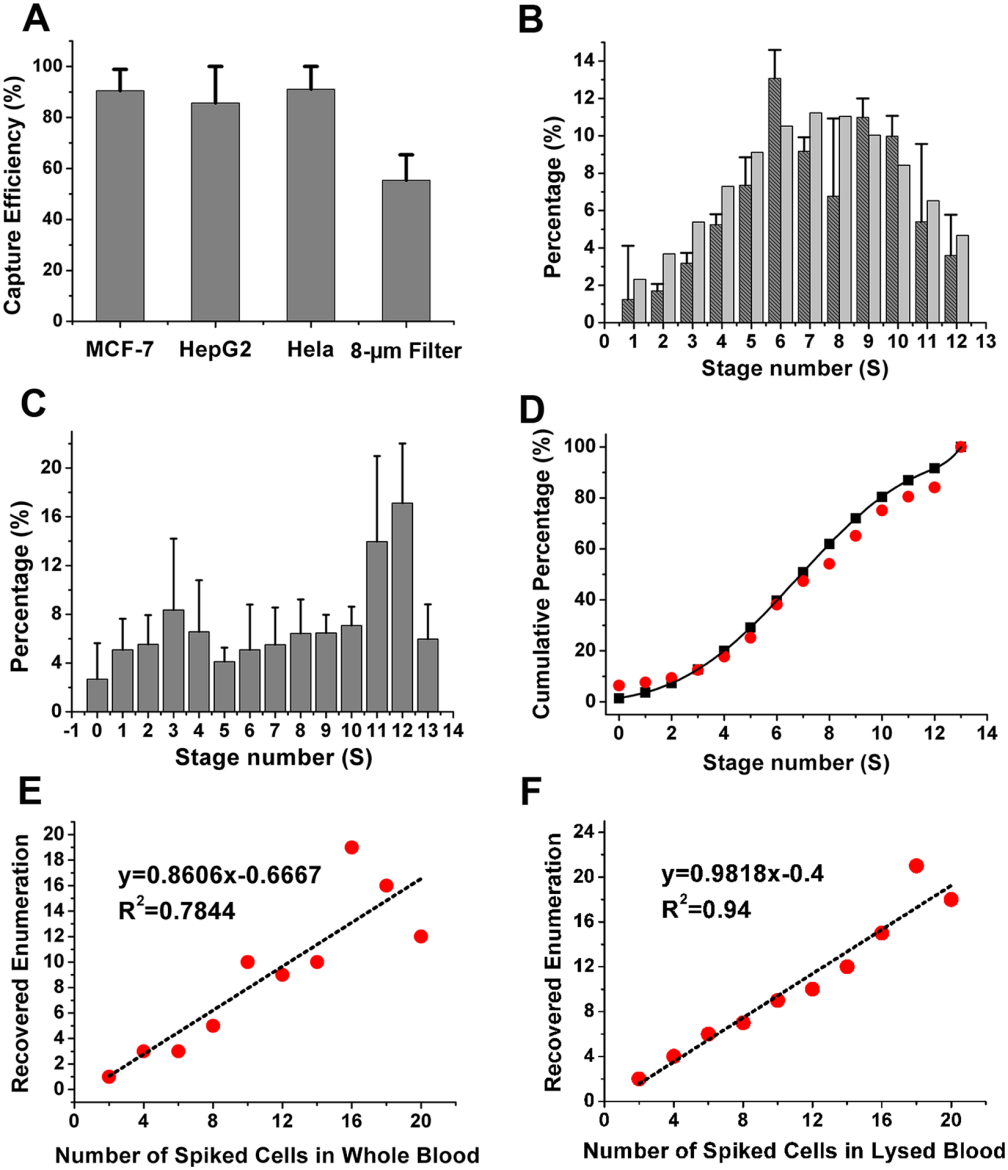
Percentage distribution of CTCs retained at individual stages. (**A**) Capture efficiencies for three different cell lines of breast (MCF-7), live (HepG2) and Hela, respectively (n = 3). CE for an 8-µm filter composed of square microposts with diameter of 20 µm for a single array. (**B**) Distribution obeys Gaussian distribution with mean value of 7.3 and standard derivation of 3.54. Dark gray is experimental values and gray is calculated ones. They are pretty fit. (**C**) Cumulative percentage distribution at various stages with 0 representing inlet and 13 as outlet. Experimental values are in black and calculated values in red (flow rate 1 ml/h). (**D**) Percentage distribution for tumor cells spiked in whole blood at various stages with mean value of 7.6. (**E**) Capture efficiencies for accurate small number of spiked MCF-7 cells in whole blood. (**F**) Capture efficiencies for accurate small number of spiked MCF-7 cells in lysed blood.

Feasibility of Ellipse filters has been tested by differential capability of tumor cells to travel through narrow capillaries. Three cell lines of MCF-7, hepatocellular carcinoma (HepG2) and Hela were utilized to rate capture efficiency (CE) capability of Ellipse filters at the flow rate of 1 ml/h. CE could reach 90.5 ± 8.4% for MCF-7, 85.7 ± 14.3% for HepG2 and 91.5 ± 8.9% for Hela (Fig. 4A), respectively, greatly satisfied with requirement of high CE of rare CTCs. The reason for trivial CE difference especially for HepG2 trivially low is that HepG2 has agglomerations phenomenon causing difficultly differentiating each cell occasionally. Performance of Ellipse filters was compared with an 8-µm filter. CE for the 8-µm filter composed of square microposts with diameter of 20 µm for a single array is 55.4% (See Supplementary Fig. 1). CE for this Ellipse filter is considerably high with the 8-µm filter. Reproducible CE indicates suitability of Ellipse filters for robust sensitivity analysis. Effectiveness of high CE is not affected by pressure experienced for tumor cells to pass through the channel. Since totally only 12 rows are arranged comparing with several hundred small-sized micropost arrays organized, CTCs avoid frequent collision while traversing through the narrow gaps, which would increases cumulative stress. Due to existence of long tunnels, there is no chance for tumor cells to get through. For our micro-ellipse 2D construction, tumor cells could regularly remain in the broader area between successive arrays before they could flow forward out. To process large sample within short time, simplified “Air Suction” is adopted to process large amount of blood sample. After red blood cells lysed (RBCL), blood sample volume greatly decreased. “Air Suction” was taken followed by absorbing the lysed sample. Therefore, the total amount is still just several hundred microliter, extremely reduced comparing with usual 2–3 ml. This approach could even be applied to 7.5 ml blood sample. Then capture procedure could be carried out and accomplished in ten minutes.

### Percentage distribution of CTCs retained by Ellipse filters at various stages

Percentage distribution of CTCs retained by Ellipse filters at each stage is assessed in Fig. 4B. Stage is defined as a zone separating two successive comb-like filters. Tumor cells are mostly confined in stages. Different deformability of tumour cells would pass through distinct levels of the gradual filter. Tumor cells appearing from downstream have stronger deformability, more invasiveness and severer malignancy in contrast to those in the upstream^31^. Deformability could especially be noted when tumor cells stuck in the middle of the tunnel. Perform three individual experiments and enumerate cell numbers at each stage, respectively. It could be deduced that percentage obeys Gaussian distribution of *μ *± *σ* = 7.3 ± 3.544 (mean value *μ* and standard derivation *σ*), corresponding to the gap spacing of first 7 µm in Ellipse filters where the most efficient percentage happens (See Supplementary for steps). Further, every stage is valid in capturing contributing to total capture partially. From Gaussian theory, probability density function is defined as $f(n,\mu ,\sigma )=\frac{1}{\sqrt{2\pi }\sigma }\exp (-\frac{{(n-\mu )}^{2}}{2{\sigma }^{2}})$, where n is stage number starting from 0 to 13. With *μ* = 7.3 and *σ* = 3.544, percentage at various stages could be calculated theoretically^35,36^ (See Supplementary). Cumulative percentage of CTCs remained at various stages is shown in Fig. 4. Dark gray and red are experimental values, and light gray and black are theoretical ones, respectively. Experimental results are consistent with theoretical calculation. Mean value has been altered to 7.6 (σ = 3.62) with same amount of MCF-7 spiked in whole blood (Fig. 4C). Complex blood constituents retard the optimal capturing position. Thus, cells distribution in various stages of the gradual filter due to different malignancy to pass through distinguishing levels of filters was evaluated.

### Tumor cell capture from artificial lysed, diluted and whole blood

Artificial patient blood was made to validate utility of Ellipse filters. Fluorescently labeled tumors cells were spiked into whole or lysed blood before using clinical samples. 2 to 20 MCF-7 cells were mixed with 1 ml whole blood or lysed blood to perform series of experiments. Those rare number cells were enumerated accurately with single fine-needle aspirates through absorbing the separated cells one followed next from diluted cell suspension, and then blowing out. An attractive result from straight best fitted lines was obtained (Fig. 4), better than 85% for whole blood and over 98% for lysed blood. CE for whole blood is relatively lower due to steric hindrance of RBCs. For whole blood, captured tumor cells experience much colliding and lashes when billions of RBCs were delivered out, posing robustness and reproducibility challenges to any microfluidic chips. Number of cells used in this assay is close to number of CTCs in real patient blood specimen. Highly-sensitivity ensures success of subsequent tests.

**Figure 5:**
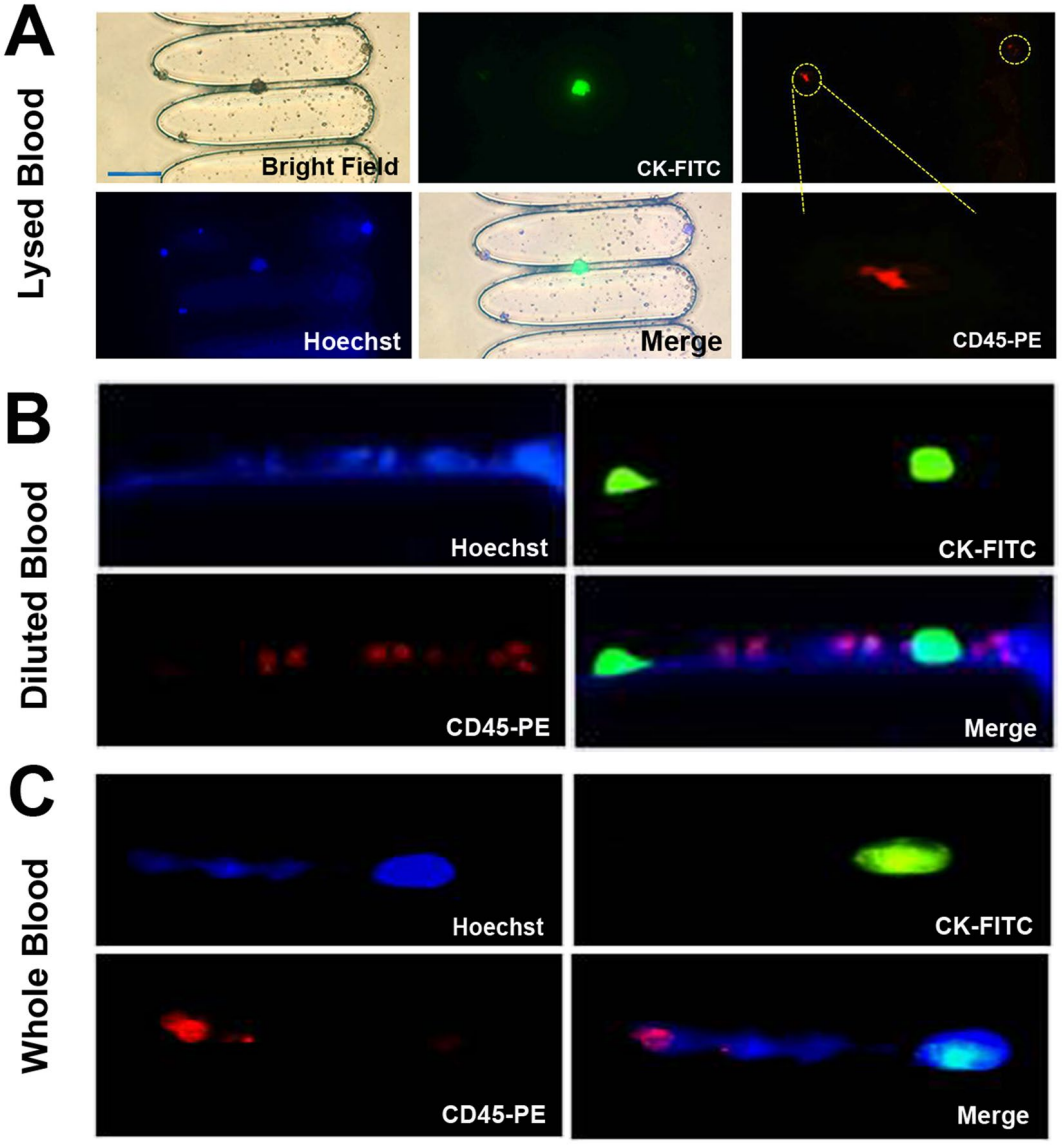
On-chip staining and fluorescence images of isolated tumor cells (green) and leukocytes (red) stained with Hoechst, CK-FITC and CD45-PE for (**A**) artificial lysed patient blood (**B**) artificial diluted patient blood (**C**) artificial whole patient blood. Scale bar = 50 µm.

To further evaluate Ellipse filters, one hundred MCF-7 cells were spiked into lysed, diluted and whole blood, respectively. After RBL, 1 ml PBS was introduced into Ellipse filters after CTCs were captured to get rid of non-specific adhesion of background cells. Captured cells were identified through staining on the chip with Hoechst, cytokeratin-FITC (CK-FITC), and CD45-PE antibodies. Figure 5 depicted MCF-7 cells with green fluorescence stuck in the channel organized by elliptical micropillars with few red leukocytes, approximately one CTC surrounded by three White blood cells (WBCs) (Fig. 5A). Tumor cell deformability is notable in the narrow constriction during passage. For large tunnels, tumor cells perhaps would occasionally remain inside instead of wandering between two neighboring arrays. Those phenomena are in accordance with our prediction.

In Fig. 5B, for artificial diluted patient blood, morphologic characteristics exhibited by two green MCF-7 tumor cells could be distinguished from several red contaminating WBCs. This is not comparable to lysed blood, but facile detection and characterization still could be performed.

For artificial whole patient blood, isolation is presumably impossible for Ellipse filters. However, large cellular sized tumor cells still could be distinguishable amidst few WBCs (Fig. 5C). Therefore, High-sensitivity has trivial influence on purity.

From illustration above, we could draw a conclusion that except few enumerable WBCs unavoidably captured on Ellipse filters, CTCs isolation could satisfactorily meet clinical demand for lysed, diluted and whole blood.

### Cell viability

**Figure 6:**
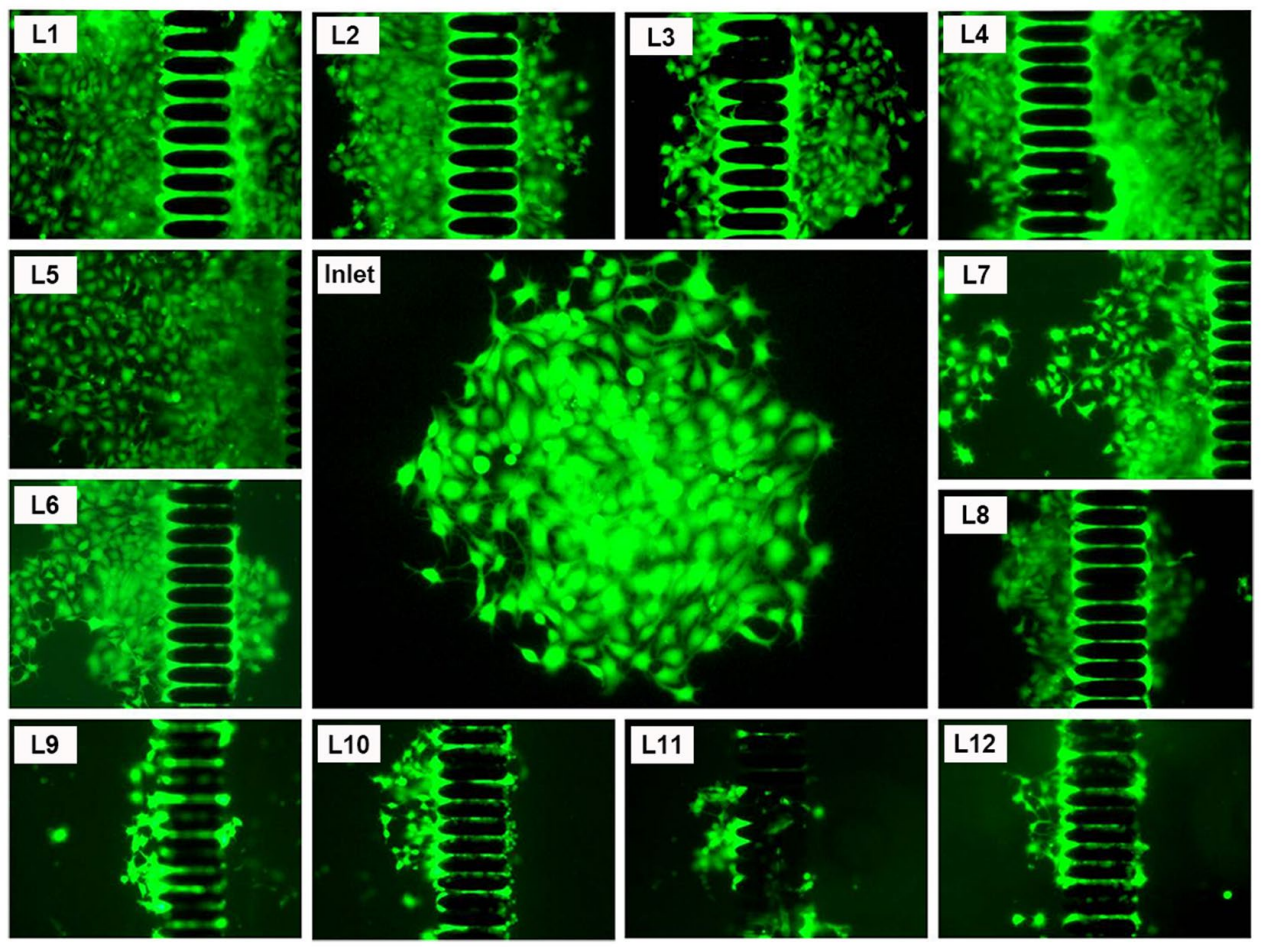
Fluorescence images of re-cultured MCF-7captured on micro-ellipse filters from L1 to L12 including inlet after 6 days.

Viability has critical significance for subsequent genetic analysis especially in clinic diagnosis. Elliptical micro-structures reduce shear stress force and gradually narrower gap spacing simplifies release procedure. Broad stages greatly mitigate compressive forces in capturing and ensure viability of released cells. After spiking MCF-7 cells into whole blood, viability is determined to be 96 ± 4.6%. Also we expand the tumor cells released after captured and cultured them at different times. The ability of the isolated cancer cells to propagate is not compromised by the capturing process (See Supplementary Figs 3 and 4). On-Chip culture after capture also could be performed (Fig. 6).

### CTCs capture in clinical patient samples of breast and non-small cell lung cancers

**Figure 7:**
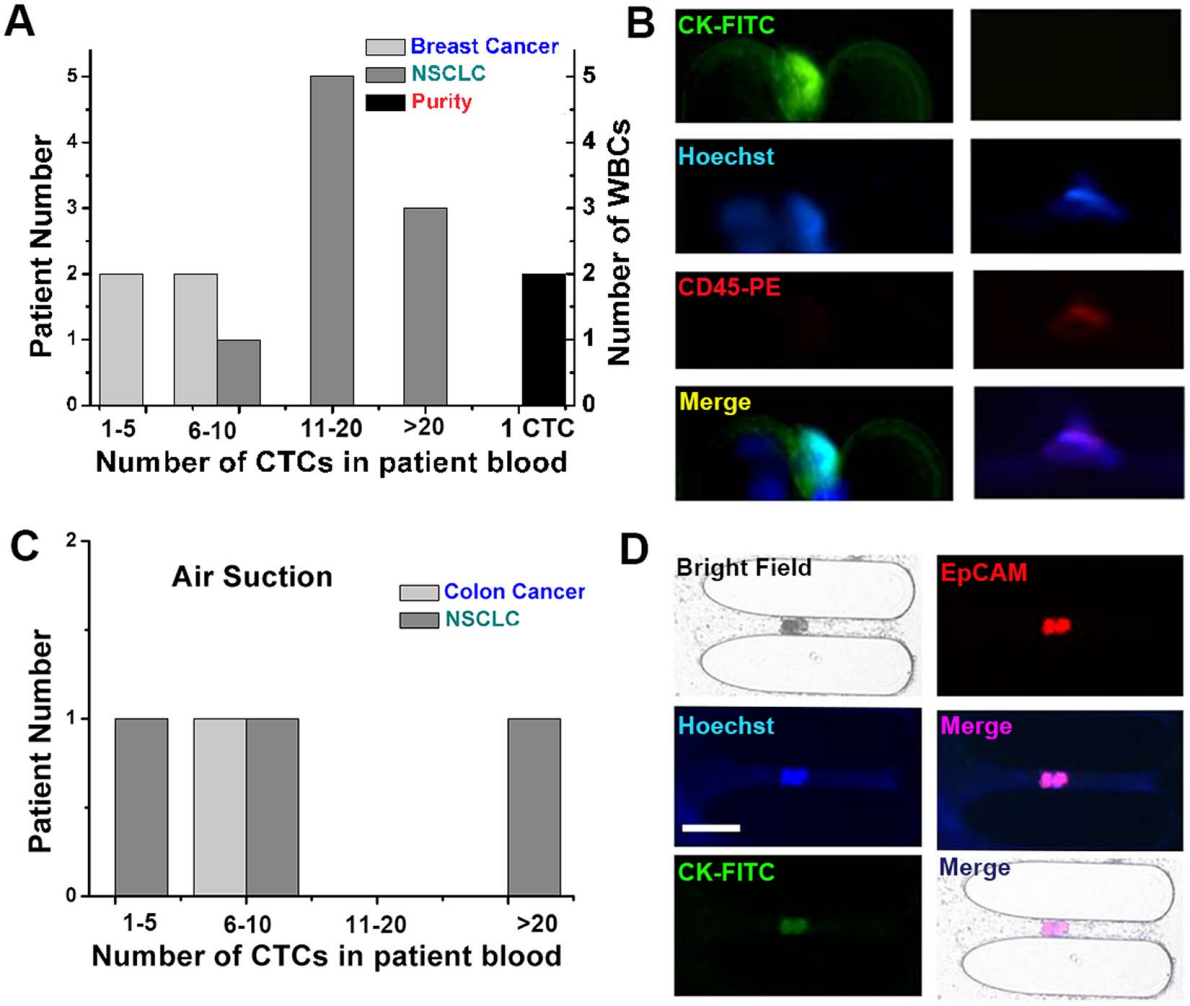
Ellipse filters validation with patient samples. (**A**) Clinical performance of Ellipse filters showing CTCs detected from patients with breast cancer and lung cancer. (**B**) Immunofluorescent staining of a breast CTC recovered showing nucleus, cytokeratin, absence of CD45 and a merged field, and similar staining for leukocytes. (**C**) Clinical performance of Ellipse filters showing CTCs detected from patients with colon cancer and lung cancer. (**D**) Immunofluorescent staining of two NSCLC CTCs recovered. Scale bar = 50 µm.

To clinically evaluate Ellipse filters, blood samples from four metastatic breast cancer patients and nine non-small cell lung cancer patients are utilized to perform assays. CTCs were tested positive for the 2–3 ml blood samples of all the patients with this Ellipse filters. Patient number involved in Ellipse filters performance evaluation corresponding to number of CTCs detected is illustrated in Fig. 7. CTCs are Hoechst+/CK+/CD45− recognized with blue and green fluorescence and WBCs are Hoechst+/CK−/CD45+ distinguished with blue and red fluorescence. Ellipse filters has been clinically assessed through capturing CTCs from four metastatic breast cancer patients and nine non-small-cell lung (NSCLC) cancer patients. With “Air Suction”, we tested one colon patient blood and three NSCLC patients. Capture could be accomplished in ten minutes. CTCs could be identified with Hoechst+/EpCAM+/CK+/CD45−. From Fig. 7D, it could be seen CTCs are cluster with red fluorescence. No CTCs has been found in health controls. For all seventeen patient samples, CTCs were detected positive in all samples with 4 patients having more than 20 CTCs. For both artificial patient blood with tumor cells spiked into the blood and clinical patient assays, purity is one CTC among few WBCs. From those fluorescence images, it could be obviously shown. This statistic result of Fig. 7A for purity derives from experimental clinical tests. Red blood cells have no effect on recognition of CTCs due to no nucleus especially after RBCL. We have demonstrated validity of this Ellipse filters in capturing and segregating CTCs in both artificial and patient blood samples.

## Conclusions

We have demonstrated unique, versatile micro-ellipse gradual filters for capturing circulating tumor cells high efficiently, allowing separation of individual population of tumor cells from heterogeneous samples. Relatively long micro-ellipse structures function as slender tunnels to block entrance of tumor cells reducing shear stress and damage, and enhancing viability. Based on physical separation of size and deformability, validity has been precisely verified in both cancer cell lines and clinical breast patient and lung patient blood specimens. Although flow rate is limited, capture efficiency is robust, rigorous and reproducible. RBCL, single fine needle aspirates, flushing and “Air Suction” combined would ensure large sample processing accomplished shortly with high purity. It could be potentially applied to CTCs product. Next step, CD45 WBCs depletion would be processed to further enhance purity and anti-cancer assays for on-chip cultured tumor cells could be conducted. It is a rigorous and sensitive analytical device for detecting, segregating and enriching CTCs, whose prospective in enumeration of CTCs in clinical samples could be expected as a diagnostic tool such as drug response and therapeutic analysis.

## Materials and Methods

### Ethics Statement and clinical sample measurement

Blood samples were obtained from healthy donors and metastatic breast and lung cancer patients in Peking University Third Hospital. The clinical sample collection was carried out in accordance with the guidelines and protocols approved by Peking University Third Hospital Medical Science Research Ethics Committee. Informed consent was obtained from all patients prior to participation in the study. Within five months before October 2016, totally 17 patient blood samples with metastatic breast cancer and non-small-cell lung (NSCLC) cancer were collected to perform clinical CTCs experiments. Among them, four are breast cancer and nine are lung cancer. All blood samples (A volume of 2–3 mL for each sample) were collected into EDTA-contained vacutainer tubes and were processed within 5 h.

### Fabrication

The size of the microfluidic chip is less than a glass microscope. The structure of the microfluidic chip is created using lithography technology. Patterns of the microstructure are drawn to produce a high resolution transparency optical photomasks. Inverse versions of the microstructures are fabricated on a silicon wafer through the following. The silicon wafer is spin-coating with a 7 µm thick AR-N 4450-10 (ALLRESIST GmbH, Germany), soft baking, UV light exposure and then post exposure baking. After developing, a silicon master pattern with the micro-structure was generated. Through casting a liquid Polydimethylsiloxane (PDMS, Sylgard 184, Dow Corning) in a monomer to curing agent ratio of 10:1, against the master and baking in an oven for 1 h at 80 °C, PDMS structure was fabricated with inlet and outlet (1.0 mm) punched. Treated with a High Frequency Generator (Electro-Technic Products, Inc, Chicago, IL), PDMS structure was bonded to a glass slide after thoroughly ultrasonic cleaning.

### Microfluidic system

The microfluidic system consists of ten microfluidic chips of micro-ellipse filters, plastic tubes, syringes and syringe pumps. A syringe pump was used to load cell suspension into the chip. The syringe attached to the pump was connected to inlet of the chip through plastic tubing as shown in Fig. 3. An inverted phase contrast fluorescence microscope (Nikon, Japan) equipped with a high speed CCD camera (Nikon, Japan) was used to observe.

### Cell Culture

MCF-7 cells (human breast adenocarcinoma) and HepG2 cells (hepatocellular carcinoma) were provided by Tianjin Medical University Cancer Institute and Hospital. Hela cells were offered by Peking University Third Hospital. Cells were cultured in Dulbecco’s Modified Eagle Medium (DMEM) (HyClone, USA) medium supplemented with 10% fetal bovine serum (FBS) (GIBCO, USA) and 1% penicillin-streptomycin (Ying Reliable biotechnology, China) and incubated in a humidified atmosphere at 37 °C with 5% CO atmosphere. When cell lines were grown as adherent monolayers to 95% confluence, they were detached from the culture dishes with 0.25% Trypsin solution. The cell suspension was diluted to obtain a desired cell concentration using a hemocytometer. Cells were labeled by Calcein AM (BIOTIUM, USA) and Hoechst (Molecular Probes, Solarbio Corp., China). Observation of CTCs was used an inverted phase contrast fluorescence microscope (Nikon, Japan) equipped with a high speed CCD camera (Nikon, Japan) and counted by using hemocytometer to around 100 cells in 1 ml PBS containing 1% BSA and 0.05% tween-20.

### Capture efficiency calculation and percentage distribution

The cell suspension was injected into microfluidic system as shown in Fig. 3. Two important characteristic parameters are defined as following to evaluate performance of Ellipse filters; i.e., capture efficiency (CE) = [(captured CTCs)/(captured + escaped CTCs)] × 100% and capture purity = [(captured CTCs)/(captured CTCs +captured WBCs)] × 100%. Number of captured cells was counted from fluorescence images from one stage to another to determine their distribution and its total number of captured cells.

After cells were fluorescently labeled with Calcein AM (BIOTIUM, USA) and Hoechst (Molecular Probes, Solarbio Corp., China), they was counted with a hemocytometer to around 100 cells in 1 ml in 1 × PBS containing 1% BSA and 0.05% tween-20. An extremely slim glass needle pulled by a micropipette puller with aspirator tube assemblies for calibrated microcapillary pipettes (Sigma-Aldrich), was used to get a desired small number of tumor cells (from 0 to 20) mixed with whole blood or lysed blood. The number of captured cells was counted from fluorescence images from one stage to another to determine their distribution. Count total number of captured cells, we should be able to get percentage distribution.

### RBCs Lysis

Red blood cells (RBCs) were removed from whole blood using Red blood cell lysis buffer (Solaibao, Beijing). It was treated gently without any damage to tumor cells and white blood cells. Operations were performed on ice. RBCs lysis was gently processed before capture, and had no effect on CTCs. The flow rate used for flushing was 1 ml/h. After RBCs Lysis, only WBCs and CTCs left. Isolation would keep every CTCs and numerable WBCs. With flushing, CTCs still remained including countable WBCs. Thus, clogging problem has been greatly mitigated.

### Artificial Patient Blood and Patient CTCs Analysis

Small limited number of unlabelled tumor cells spiked into the lysed, diluted and whole blood, respectively. Those artificial patient bloods were processed through the chip followed by washing with PBS, fixing and permeabilization. Anti-Cytokeratin (BD Biosciences) and anti-CD45 (BD Biosciences) and Hoechst applying in 1% bovine serum albumin were utilized for all samples. After washing, then it is ready for microscopic imaging. Normal blood samples and clinical patient samples of metastatic breast, colon and non-small cell lung cancers are offered by Peking University Third Hospital under approval. Same procedure including staining was followed for the patient CTCs analysis.

### Cell Viability Assay

MCF-7 cells were harvested, spiked into whole blood, and flowed through the micro-ellipse filters at 1 ml/h. The live/dead reagent consisting of calcein AM and ethidium homodimer-1 (Invitrogen – Life Technologies, Inc.) prepared specifically according to instruction of the manufactures. Number of alive and apoptosis cells were counted respectively to determine viability.

## Electronic supplementary material

Supplementary Information
