# Author Correction: Highly-sensitive capture of circulating tumor cells using micro-ellipse filters

**DOI:** 10.1038/s41598-018-22955-w

**Published:** 2018-03-22

**Authors:** Hongmei Chen, Baoshan Cao, Bo Sun, Yapeng Cao, Ke Yang, Yu-Sheng Lin

**Affiliations:** 1 Institute of Semiconductors, Chinese Academy of Sciences, Beijing, 100083 China; 2 Division of Nanobionic Research, Suzhou Institute of Nano-Tech and Nano-Bionics, Chinese Academy of Sciences, Suzhou, Jiangsu 215123 China; 3 Department of chemotherapy and radiation sickness, Peking University Third Hospital, Beijing, 100191 China; 4 Physics Department, University of Massachusetts Lowell, Lowell, Massachusetts 01854 USA

Correction to: *Scientific Reports* 10.1038/s41598-017-00232-6, published online 04 April 2017

Hongda Chen did not contribute directly to this study and has been removed from the author list. Correspondence and requests for materials should be addressed to H.C. (email: hongmeichen@semi.ac.cn).

The Acknowledgements section now reads:

“Many thanks are given to Nanjing University contributed to idea of “Air Suction”, simulation and important discussion. Great acknowledgement is given to Prof. Hongda Chen from Chinese Academy of Sciences for his great effort in guiding the whole project, carefully detailed instruct progress of this research and Nobel decline to add his name as coauthor. Dr. Baoshan Cao from Peking University Third Hospital carefully provided clinical patient samples. Yu-Sheng Lin provided suggestion in revising the manuscript. Zhaoxin Geng from Chinese Academy of Sciences proposed 10-channel pump. Graduate students Hongsheng Gao, Tengfei Wang, Chunfei Hu and Guoqing Song from Chinese Academy of Sciences got involvement in discussion. This research work was supported by the Major State Basic Research Development Program of China (973 Program) (Grant No. 2011CB933102) and National Natural Science Foundation of China (Key Program: 61335010).”

The Author Contributions section now reads:

“Hongmei Chen contributed many works in designing the chip, and performing experiments including clinical experiments, and writing the manuscript. Baoshan Cao get involved in the clinical experiment. Yapeng Cao designed mask for fabrication. Bo sun provided and cultivated the cells. Yu-Sheng Lin, Ke Yang revised the manuscript. All authors contributed to scientific discussion and critical revision of the article.”

These errors have now been corrected in the PDF and HTML versions of the Article, as well as the Supplementary Information file.

