# Supplementary material for: Highly-sensitive capture of circulating tumor cells using micro-ellipse filters
Source: Sci Rep. 2017 Apr 4;7:610. doi: 10.1038/s41598-017-00232-6 (PMC5428045; doi:10.1038/s41598-017-00232-6)
Supplement: Supplementary file 1 — Supplementary Information [file 41598_2017_232_MOESM1_ESM.doc]

**Highly-sensitive capture of circulating tumor cells using micro-ellipse filters**

Hongmei Chen^1, 3*^, Baoshan Cao^2^, Bo Sun^1^, Yapeng Cao^1^, Ke Yang^4^ and Yu-Sheng Lin^3*^

*^1^Institute of Semiconductors, Chinese Academy of Sciences, Beijing, 100083, China*

*^2^Department of chemotherapy and radiation sickness, Peking University Third Hospital, 100191, China*

*^3^Division of Nanobionic Research, Suzhou Institute of Nano-Tech and Nano-Bionics, Chinese Academy of Sciences, Suzhou, Jiangsu, 215123, China*

*^4^Physics Department, University of Massachusetts Lowell, Lowell, Massachusetts, 01854, USA*

* To whom correspondence should be addressed. E-mail: hongmeichen@semi.ac.cn

**Supporting Materials**

In order to find out the optimal gap spacing between micro-elliptical microposts, we firstly design a simple microfluidic chip to test efficiency of 8 μm gap spacing. Smallest microposts in the third row are circles or squares with diameter or side of 20 µm in length. As shown in Fig.S1, when tumor cells MCF-7 coated with magnetic beads entered the area in front of the microposts at 1 ml/h, majority of them were extensively sorted. However, from Fig.S1, it could be seen that several size amplified tumor cells tend to get across to right side of microposts wall, indicating captured tumors cells still had high opportunities to escape away. Therefore, 8 µm gaps are still not perfect enough to capture every CTC at any situations especially at high flow rate or for small sized tumor cells such as non small-cell lung cancer (NSCLC). A long and narrow aperture could be developed in case seized CTCs passage through.


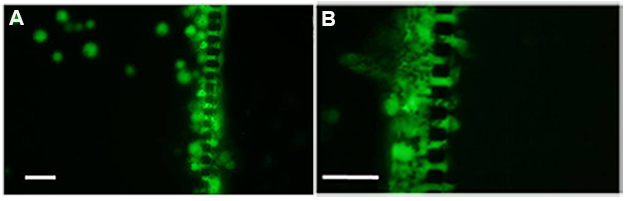


**Figure S1.** MCF-7 cells coated with magnetic immune-beads captured by a simple microfluidic chip with gaps of 8 µm (A) MCF-7 cells were captured on both sides of the microposts.Scale bar = 100 µm. (B) MCF-7 cells had tendency to pass over 8 µm gaps (Inlet from left). Scale bar = 100 µm.

**Table** **1**.Current methods for CTCs isolation (capture efficiency (CE), capture purity (CP))

| Author | Structure | Antibody/Size | Clinical samples/Cell line | CE | CP | Viability | Throughput |
| --- | --- | --- | --- | --- | --- | --- | --- |
| Nagrath et al[^1^](#_ENREF_1) | CTC-chip | Anti-EpCAM | 116 metastatic lung, prostate, pancreatic, breast and colon cancer | 65% | 50% |  | 1-2 ml/h |
| Stott et al[^2^](#_ENREF_2) | Herringbone-chip (HB-Chip) | Anti-EpCAM | 15 patients at various stages of treatment for metastatic prostate cancer | 91.8% | 14.0% | 95% | 1.2 ml/h |
| Cari Launiere et al[^3^](#_ENREF_3) | Patterning regions of alternating adhesive proteins | Anti-EpCAM and E-selectin | MCF-7 and HL-60 | 90.1% | 84.7% | --- | 8 µl/min |
| Hyeun Joong Yoon et al[^4^](#_ENREF_4) | A grapheme oxide nanosheets (flower-shaped gold patterns) | Anti-EpCAM | Metastatic breast cancer (n=7), early-stage lung cancer (n=4) and metastatic pancreatic cancer | 94.2% | --- |  | 1.0 ml/h |
| Hyeun Joong Yoon et al[^5^](#_ENREF_5) | Tunable Thermal-Sensitive Polymer-Graphene Oxide Composite | Anti-EpCAM | 10 metastatic breast cancer patients and 3 pancreatic cancer patients | 84.93-95.21% | --- | 91.68% | 1.0 ml/h |
| Murlidhar et al[^6^](#_ENREF_6) | OncoBean chip | Anti-EpCAM | Pancreatic (n=2), breast (n=2) and lung (n=2) cancer patients | 80% | 390-740 cells/mL | 93% | 10 ml/h |
| A Fatih Sarioglu et al[^7^](#_ENREF_7) | A CTC Cluster-Chip | 12 μm | MCF-7,MDA-MB-231,MCF10A-LBX1,breast cancer (n=27), melanoma (n=20) and prostate cancer (n=13) | 99% | --- | --- | 2.5 ml/h |
| Chung et al.[^8^](#_ENREF_8) | MSS chip and a size-sorter | 5 μm | HT29 in RBC-lysed blood  --- | >80% | --- | >10^3^ | 3 ml/h |
| Jakub Chudziak et al[^9^](#_ENREF_9) | Parsortix system (Parsortix) | 10 μm | HT29, H2009 and DMS114 | 78% | --- | --- | 2 ml/h |
| Hur et al.[^10^](#_ENREF_10) | expansion-contraction trapping reservoir | --- | Hela, MCF-7  --- | ~43%(Hela) ~20%(MCF-7) | 85% | --- | --- |
| Lin et al.[^11^](#_ENREF_11) | a parylene membrane filter-based microdevice | 8 μm | RT4, T24, J82, HT-1080, LNCaP,MCF-7,MDA-MB-231  --- | >90% | --- | --- | >225 ml/h |
| Tilman Todenhofer[^12^](#_ENREF_12) | Microfluidic funnel rachets | 8 μm | 50 prostate cancer patients | 98% | 99% | >95% | Oscillatory pressure of 7 kPa |
| McFaul et al.[^13^](#_ENREF_13) | microfluidic rachets | 12-2 μm | L1210 mouse lymphoma cells | 98% | 99% | --- | 9000 cells h^-1^ |
| Present paper | Micro-Ellipse filters | 18-5 μm | MCF-7, Hela, HepG2  Metastatic breast cancer (n=4), colon (n=1) and NSCLC (n=12) | >90% | --- | 90% | 1~3ml/h |


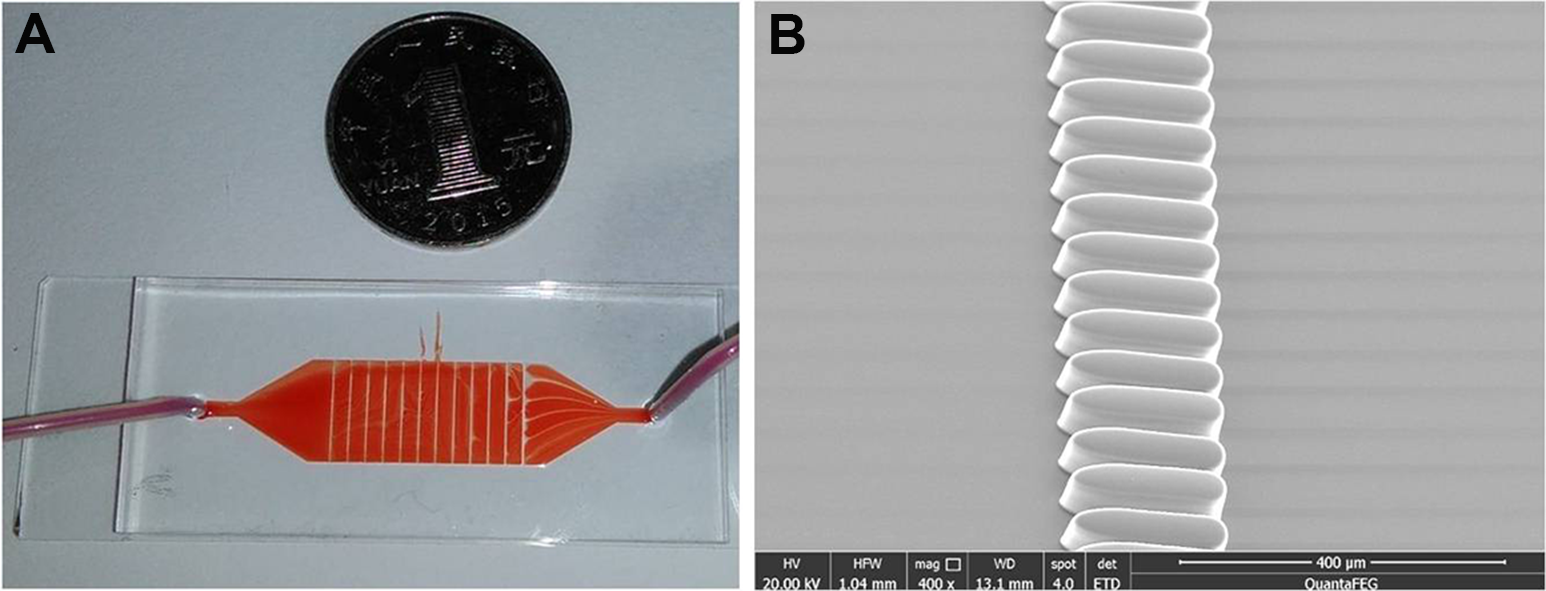


**Figure S2.** (A) Ellipse filters in blood assays. (B) Scanning electron micrograph (SEM) image of the microposts array.

Calculation for$\mu$ $\pm$ $\sigma$=7.3$\pm$3.544

Firstly, perform three individual experiments and count cell numbers at various stages, respectively. Secondly, percentage at each stage should be able to be got through divided by total number captured by each chip. Thirdly, average three percentages at each stage, and then we should be able to get average percentage and cumulative percentage. Fourthly, using those 12 average percentage values times 1 to 12, respectively and average again, we should be able to get mean value corresponding to stage position located at the chip. For example, $\mu$=7.3 in our calculation is the gap spacing of first 7 µm in Ellipse filters where the most efficient happens.

Use mean value minus 1 to 12 and square them, respectively. Add those 12 values together, and then average them followed by square root. We should be able to get standard derivation such as 3.544.

According to Gaussian theory and probability density function,$f(n,\mu,\sigma)=\frac{1}{\sqrt{2\pi}\sigma}\exp(-\frac{(n-\mu)^{2}}{2\sigma^{2}})$, we replace $\mu$=7.3 ,$\sigma$=3.544, and stage number n from 0 to 13. Put those thirteen group numbers into the formula and times 100, we should be able to get probability at each stage. For example,

$f(1,7.3,3.544)=\frac{1}{\sqrt{2\pi}*3.544}\exp(-\frac{(1-7.3)^{2}}{2*(3.544)^{2}})$, $f(1,7.3,3.544)$=0.023,

Probability at 1st stage is P (1) =2.32%. Cumulative probability at 1st stage is C (1) =2.32%. With the same way,

$f(2,7.3,3.544)=\frac{1}{\sqrt{2\pi}*3.544}\exp(-\frac{(2-7.3)^{2}}{2*(3.544)^{2}})$, $f(2,7.3,3.544)$=0.0368,

Probability at 2nd stage is P (2) =3.7%. Cumulative probability at 2nd stage is C (2) =2.32%+3.68%= 6%. Following this way, probability distribution and cumulative probability distribution at various stages should be able to be got.

Table 2 Probabilities at various stages of Ellipse filters

| **Stage Number** | **Probability**  **density** | **Cumulative probability density** | **Probability** | **Cumulative probability** |
| --- | --- | --- | --- | --- |
| 1 | 0.0232 | 0.0232 | 2.32 | 2.32 |
| 2 | 0.0368 | 0.06 | 3.68 | 6 |
| 3 | 0.0539 | 0.1139 | 5.39 | 11.4 |
| 4 | 0.073 | 0.1869 | 7.3 | 18.7 |
| 5 | 0.0912 | 0.2781 | 9.12 | 27.8 |
| 6 | 0.1053 | 0.3834 | 10.5 | 38.3 |
| 7 | 0.1122 | 0.4956 | 11.2 | 49.6 |
| 8 | 0.1104 | 0.606 | 11 | 60.6 |
| 9 | 0.1003 | 0.7063 | 10 | 70.6 |
| 10 | 0.0842 | 0.7905 | 8.42 | 79.1 |
| 11 | 0.0653 | 0.8558 | 6.53 | 85.6 |
| 12 | 0.0467 | 0.9025 | 4.67 | 90.3 |


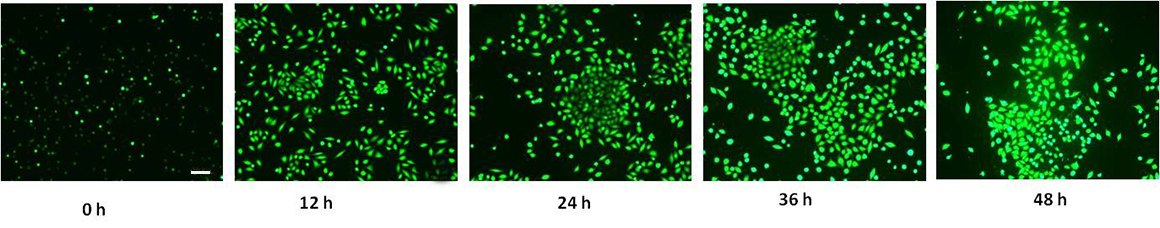


**Figure S3.** Fluorescence images of cultured MCF-7. Cells released from the chip immediately, 12 h, 24 h, 36 h and 48 h. Scale bar = 100 µm.


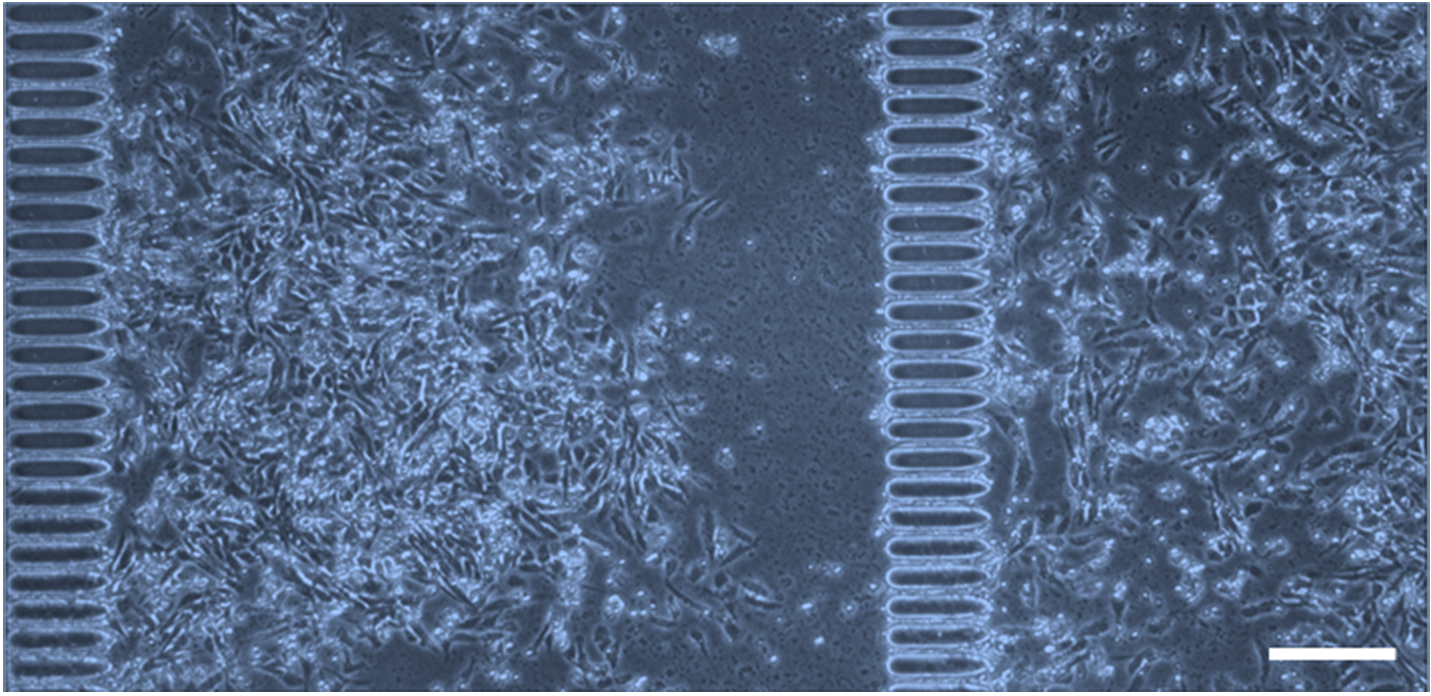


**Figure S4.** On-chip culture of lung cancer cells A549 after capture. Scale bar = 100 µm.

**References**

1. Nagrath, S. et al. Isolation of rare circulating tumour cells in cancer patients by microchip technology. *Nature* **450**, 1235-U1210 (2007).

2. Stott, S.L. et al. Isolation of circulating tumor cells using a microvortex-generating herringbone-chip. *Proc. Natl. Acad. Sci. U. S. A.* **107**, 18392-18397 (1072010).

3. Launiere, C. et al. Channel surface patterning of alternating biomimetic protein combinations for enhanced microfluidic tumor cell isolation. *Analytical chemistry* **84**, 4022-4028 (2012).

4. Yoon, H.J. et al. Sensitive capture of circulating tumour cells by functionalized graphene oxide nanosheets. *Nature nanotechnology* **8**, 735-741 (2013).

5. Yoon, H.J. et al. Tunable Thermal-Sensitive Polymer-Graphene Oxide Composite for Efficient Capture and Release of Viable Circulating Tumor Cells. *Advanced materials* (2016).

6. Murlidhar, V. et al. A Radial Flow Microfluidic Device for Ultra-High-Throughput Affinity-Based Isolation of Circulating Tumor Cells. *Small* **10**, 4895-4904 (2014).

7. Sarioglu, A.F. et al. A microfluidic device for label-free, physical capture of circulating tumor cell clusters. *Nature methods* **12**, 685-691 (2015).

8. Chung, J. et al. Rare cell isolation and profiling on a hybrid magnetic/size-sorting chip. *Biomicrofluidics* **7**, 9 (2013).

9. Chudziak, J. et al. Clinical evaluation of a novel microfluidic device for epitope-independent enrichment of circulating tumour cells in patients with small cell lung cancer. *The Analyst* **141**, 669-678 (2016).

10. Hur, S.C., Mach, A.J. & Di Carlo, D. High-throughput size-based rare cell enrichment using microscale vortices. *Biomicrofluidics* **5**, 10 (2011).

11. Gogoi, P. et al. Development of an Automated and Sensitive Microfluidic Device for Capturing and Characterizing Circulating Tumor Cells (CTCs) from Clinical Blood Samples. *PloS one* **11**, e0147400 (2016).

12. Todenhofer, T. et al. Microfluidic enrichment of circulating tumor cells in patients with clinically localized prostate cancer. *Urologic oncology* (2016).

13. McFaul, S.M., Lin, B.K. & Ma, H.S. Cell separation based on size and deformability using microfluidic funnel ratchets. *Lab Chip* **12**, 2369-2376 (2012).
